# Screening Dementia in the Outpatient Department: Patients at Risk for Dementia

**DOI:** 10.1155/2014/138786

**Published:** 2014-12-08

**Authors:** Shu-Yu Tai, Shu-Wan Huang, Chia-Ling Hsu, Chiu-Hsien Yang, Mei-Chuan Chou, Yuan-Han Yang

**Affiliations:** ^1^Department of Family Medicine, Kaohsiung Municipal Ta-Tung Hospital, Kaohsiung City 80145, Taiwan; ^2^Department of Family Medicine, Kaohsiung Medical University Hospital, Kaohsiung Medical University, Kaohsiung City 80708, Taiwan; ^3^Department of Neurology, Kaohsiung Municipal Ta-Tung Hospital, Taiwan; ^4^Department of Neurology, Kaohsiung Medical University Hospital, Kaohsiung 80708, Taiwan; ^5^Mentality Protection Center, Fo Guang Shan Compassion Foundation, Kaohsiung City 80050, Taiwan; ^6^Department of and Master's Program in Neurology, Faculty of Medicine, Kaohsiung Medical University, Kaohsiung City 80708, Taiwan

## Abstract

The targeted screening for individuals at the risks of having dementia would be crucial to the further public health issues for dementia. This study aimed to conduct a screening study in an outpatient department of a regional hospital to screen people who were at risk of developing comorbid dementia. Patients who visited Kaohsiung Municipal Ta-Tung Hospital (KMTTH) clinics during the period from June 1, 2013, to May 31, 2014, were invited to participate in this screening voluntarily. The trained interviewer collected all participants' demographic characteristics and used the instrument of ascertainment of dementia 8 (AD8) to find out suspected dementia ones. The result showed a higher ratio (24.1%) of suspected dementia in the outpatient department of a hospital, 500 out of 2017 subjects, than that in the general population. The median (interquartile range) age was significantly higher in the suspected dementia participants (70, (62, 77)) compared to that in nonsuspected dementia ones (65, (60, 73)), and the probability of suspected dementia was significantly increasing with age (*P* < 0.001). Instead of screening dementia in general population, screening people at the risk of dementia could be the practicable and important issues in the care of dementia.

## 1. Introduction

Dementia is a major public health problem related to the aging population in developed countries. The aged population has increased rapidly in Taiwan, from 10.74% in 2010 to 11.53% in 2013 and is estimated to reach 20% in 2025 [1]. With the rapidly increasing aged population, the global prevalence of Alzheimer's disease (AD) is expected to dramatically increase by 2050, with Asia estimated to account for 59% of worldwide cases [2]. The increasing prevalence of cognitive impairment in the general population emphasizes the need for early intervention and treatment.

Conducting broad dementia screening in the general population is unreasonable because the prevalence rate of dementia and related disorders among people is too low to yield satisfactory results. However, because the risk of dementia increases substantially with age, approaching 45% among Americans aging 85 and older [3, 4], the targeted screening of at-risk individuals is reasonable.

In addition, according to the World Alzheimer Report from 2013 [5], people with dementia demonstrate a high level of use regarding health services and represent a large fraction of the healthcare costs attributed to the elderly population. Moreover, patients with dementia have an average of 2 to 8 additional chronic diseases (comorbidities) [6, 7]. These chronic diseases may accelerate progression towards a state of cognitive and functional impairment that lead to increased hospital visits and result in the underdiagnosis and undertreatment of dementia [8]. Similar to other clinically complex circumstances, the presence of comorbidities in patients with chronic diseases requires the careful consideration [9] of possible conflicts between multiple treatments and recommendations for these patients [10]. Patients who visit outpatient departments are at a higher risk of developing comorbid dementia because of their multiple underlying diseases compared with those who do not [6]. In addition, previous studies have indicated that most suspected dementia patients visit primary physicians first instead of neurologists [7, 11]. Therefore, identifying a high risk of dementia in people who visit outpatient departments in all primary clinics is crucial for early diagnosis and early treatment.

Kaohsiung Municipal Ta-Tung Hospital (KMTTH) is a regional teaching hospital in Southern Taiwan, which was established and founded on January 15, 1946, and originally named the “Kaohsiung Municipal Hospital.” Currently, KMTTH has 17 clinical departments (including internal medicine, surgery, obstetrics/gynecology, pediatrics, orthopedics, ophthalmology, otolaryngology, a radiation oncology center, urology, dermatology, rehabilitation, psychiatry, family medicine, occupational medicine, dentistry, a neurology clinic, and preventive medicine) and 479 beds. On average, KMTTH serviced approximately 40,000 clinic patients, 3,500 emergency visitors, and 7,000 acute ward admission patient-days per month during 2013. As a regional teaching hospital, the service regions of KMTTH include the Cianjin, Hsingsin, and Yenche Districts with 3.88% Kaohsiung residents (107773/2778920), where the percentage of older people in the population is high and growing (≥65 years: 22.39% in these 3 districts versus 11.08% in Kaohsiung City) [12]. The targeted screening of at-risk individuals in a hospital that provides services for this aged population could be highly effective in dementia care. Patients who visited the KMTTH outpatient department were invited to participate in this study by voluntarily undergoing screening for dementia using the AD8 from June 1, 2013, to May 31, 2014. The methods and results are detailed in the following section.

## 2. Material and Methods

### 2.1. Participants and Evaluation

Patients who had visited KMTTH clinics (including the 17 clinical departments) during the period from June 1, 2013, to May 31, 2014, were invited to participate in the screening activity voluntarily. The trained interviewer collected the information of the age, sex, and living area of all participants. All procedures were approved by the Kaohsiung Medical University Hospital Institutional Review Board. All private information and information that could be used to identify the participants were not recorded during the screening process.

### 2.2. Cognitive Screening (Ascertainment of Dementia 8 (AD8))

AD8 is a brief tool used to screen dementia that was developed at Washington University in St. Louis [13] and is capable of screening extremely mild dementia in the general population; it is also used extensively in multiple countries, including Taiwan, after validation [14]. If the score of AD8 is greater than or equal to 2, the person is considered to be suspected of dementia, including extremely mild dementia [14]. The AD8 can be conducted on the informant of patients with dementia and the patients themselves with a similar discriminative rate in differentiating patients with and without dementia [15].

All the participants were interviewed face-to-face by the trained interviewer to assess their cognitive function status with the instrument of AD8. A participant was considered to be suspected of dementia with a cut-off of total AD8 score greater than or equal to 2.

### 2.3. Statistics

Data analysis was performed using SPSS software (version 12.0.1 for Windows; SPSS Inc., Chicago, IL, USA). All statistical tests were 2-tailed and *P* value of 0.05 was considered statistically significant. Wilcoxon rank sum test, a nonparametric test (*α* = 0.05), was used to compare the median (interquartile range) of age and total AD8 score between patients of suspected and nonsuspected dementia. We divided the continuous variable of age into 4 categories: not elderly (<65 y old), young elderly (65–74 y old), old elderly (75–84 y old), and “super-old” elderly patients (≥85 y old) for easy comparison [14–17]. Kruskal-Wallis test was used to compare the difference in the median of total AD8 score among the age groups of all the suspected dementia patients. The chi-square test was used to compare the proportion of each reported subitem of the AD8 and gender between the patients with suspected and nonsuspected dementia and among the age groups (<65, 65–74, 75–84, and ≥85 years) of all suspected dementia patients.

## 3. Results

We have recruited 2071 participants during their visit of clinic at the outpatient department into the statistical analysis. The medians (interquartile range) age of years and AD8 score were (66, (60, 74)) and (0, (0, 1)) the majority of the participants were female (52.4%) (Table 1). A total of 500 participants (24.1% of all the recruited participants) had suspected dementia with an AD8 score of greater than or equal to 2 (Table 1).

The median of age (*P* < 0.001) and total AD8 score (*P* < 0.001) were significantly different between the groups of nonsuspected and suspected dementia, but the distribution of sex was similar between the two groups (*P* = 0.914) (Table 2).

The ratio of reported change for each subitem of the AD8 and total AD8 score were significantly different between people with nonsuspected and suspected dementia (*P* < 0.001 for the comparison of each AD8 subitem and total AD8 score) (Table 2). In the nonsuspected dementia group, subitems AD8-8 (consistent problems with thinking and/or memory) (14.5%), AD8-2 (reduced interest in hobbies/activities were frequently reported) (7.1%), and AD8-3 (repeats questions, stories, or statements) (5.5%) were the 3 most frequently reported “changes” among the AD8 subitems (Table 2). These frequently reported changes in the nonsuspected dementia group were similar to those reported in the suspected dementia group, in which AD8-8 (84.4%), AD8-2 (48.0%), and AD8-3 (50.8%) were frequently reported (Table 2), and the frequencies were higher in the suspected dementia group compared with those in the nonsuspected dementia group.

The ratio of patients with suspected dementia significantly increased with age to 18.9%, 24.3%, 29.6%, and 47.8% among the <65, 65–74, 75–84, and ≥85 age group categories (*P* for trend < 0.001), respectively (Table 3). The distribution of sex was not significantly different among the age groups (*P* = 0.124) of all suspected dementia participants, but the AD8 score was significantly different (*P* = 0.005). The AD8 score was the highest (median, (interquartile range): 4, (2,5)) in the ≥85 age group and the lowest (median, (interquartile range): 3, (2,4)) in the <65 age group. The ratio of reported change for each subitem of the AD8 and total AD8 score were significantly different among age groups, except for AD8-2 (reduced interest in hobbies/activities) (*P* = 0.385) and AD8-5 (forgets the correct month or year) (*P* = 0.220) (Table 3). Among the subitems of the AD8 with a significant difference among age groups (AD8-1, AD8-3, AD8-4, AD8-6, AD8-7, and AD8-8), the ≥85 age group had a higher ratio of reported change compared with that of the other age groups, except for AD8-8.

## 4. Discussion

In this study, a higher ratio (24.1%) of dementia was observed in the outpatient department of KMTTH clinics compared with that in the general population (13.6% in people who underwent general screenings and 8.0% for the age-adjusted prevalence of all-cause dementia) [18, 19] and the mean age was significantly higher in the suspected dementia participants compared with that of the participants without dementia. In addition, the ratio of dementia was determined to increase rapidly with old age.

For the general population, the results of a recent walk-in screening for dementia in Taiwan [18] indicated a 13.6% ratio of dementia, which was higher than that reported in previous studies. In addition, another nationwide population-based cross-sectional survey conducted in Taiwan indicated that the age-adjusted prevalence of all-cause dementia was increasing (8.0%) and old age, female gender, and a low educational level were significant associated factors [19]. Several differences exist between these 2 studies and this study. First, the people who underwent targeted screening in this study conducted in the outpatient department may be more at risk or exhibit more morbid conditions than the people in the previous 2 studies conducted in the general population. Second, the assessment tool used was different between one of the 2 recent studies and our study. We used the AD8 as the screening instrument, which is capable of being used to screen extremely mild dementia [13–15] and is more sensitive compared with other tools used previously (e.g., the mini-mental state examination) [16, 20, 21]. However, all 3 studies have demonstrated that the estimated number of people with dementia in Taiwan during the 2010s is rapidly increasing with the increase in the aged population (the proportion of people over 65 y of age increased from 6.8% in 1992 to 11.1% in 2012) [12], although different diagnostic criteria and study designs might have contributed to the variations in the epidemiological estimates reported in these studies.

In addition, we determined that the AD8 score significantly increased with age and the “super-old” group (≥85 y) had a considerably higher proportion of participants with suspected dementia (47.8%) compared with those in the other age groups. This result was consistent with most of the previous studies that have indicated that cognitive function declines with age [22]. Furthermore, these suspected dementia patients should be monitored by their primary physicians because they will most likely develop dementia, based on the high sensitivity and specificity of the AD8 in screening dementia. Moreover, Taiwan was estimated to be an aged society (>14% elderly people) in 2017 and a “super-old” society (>20%) in 2025 according to the survey conducted by the Taiwan Alzheimer's Disease Association [23]. Therefore, detecting people who are at risk for dementia early with a highly sensitive tool is a crucial concern for healthcare units because early diagnosis may increase the beneficial effects of new therapies.

Each reported subitem of the AD8 was significantly different between the participants with suspected dementia and those without dementia, and the 3 most frequently reported AD8 subitems exhibited no significant difference (AD8-8, AD8-2, and AD8-3). However, the distribution of frequently reported AD8 subitems was different from that of the general population, in which AD8-8 (6.4%), AD8-2 (3,1%), and AD8-5 (3.0%) were the 3 most frequently reported in the group without dementia and AD8-8 (56.8%), AD8-7 (47.0%), and AD8-5 (40.9%) were the most frequently reported in the suspected dementia group [18]. Among the age groups of suspected dementia participants, the reported change of AD8 subitems was significantly different, except for AD8-2 (reduced interest in hobbies/activities) and AD8-5 (forgets the correct month or year). KMTTH is located in an urban but cultural community that contains more retired government employees and teachers, which may cause reduced fluctuation in the activities and living habits of the residents. Similarly, retired and aged people with a high education level and cultural background may meticulously arrange their schedule activities, causing them to note the date frequently. In this study, the “super-old” (≥85 years) participants all reported a higher proportion of AD8 subitems, except for AD8-8 (consistent problems with thinking and/or memory). Generally, the impairment of recent memory could be the initial presentation of extremely mild dementia, especially in people with Alzheimer's disease [24, 25]. We recruited few overall and suspected dementia participants in the “super-old” group and no clinical data on the subtype of dementia were obtained to explain this result. Therefore, further study on the various characteristics of elderly people may be required.

This study has some strength. First, this is the first study to target and screen at-risk patients in an outpatient department where they were usually neglected in a routine screening of dementia. Second, we use the AD8 as a screen tool that was capable of screening dementia at its very mild stage. However, this study has several limitations. First, the participants were not recruited using randomized sampling from the general population, which may influence the external generalization of the results. Second, the participants recruited from one single regional hospital cannot be applied to all hospitals and they may exhibit more morbid conditions than others do. Third, we had neither detailed information about comorbidities and education nor information on the further diagnosis to clarify whether he was dementia or not. However, the aim of this study was to screen at-risk people who visited the outpatient department of a hospital. The other goal of this study was to provide the primary physicians at the hospital with data on the high ratio of dementia patients who visited the outpatient department and suggest that they screen and diagnose these patients early because most dementia patients visit primary physicians instead of neurologists [7, 11].

In conclusion, we observed a higher ratio (24.1%) of dementia in the outpatient department of a hospital than in the general population, and the ratio was confirmed to increase rapidly with age.

## Figures and Tables

**Table 1:** Demographic characteristics of all the recruited participants.

	Total
Number (*N*, %)	2071, 100%
Age, years	66, (60, 74)
(median, (IQR^&^))	
<65	888, 42.9%
65–74	667, 32.2%
75–84	426, 20.6%
≥85	90, 4.3%
Female (*n*, %)	1085, 52.4%
AD8 score	0, (0, 1)
(median, (IQR^&^))	
<2	1571, 75.9%
≥2	500, 24.1%

^&^IQR: interquartile range.

**Table 2:** AD8 subitems for the nonsuspected and suspected dementia participants.

AD8 subitems	Nonsuspected	Suspected^#^	*P* value
Number	1571	500	
(*N*, %)	75.9%	24.1%	
Age, years	65, (60, 73)	70, (62, 77)	<0.001^*※*^
(median, IQR^&^)			
<65	719, 45.8%	169, 33.8%	<0.001
65–74	505, 32.1%	162, 32.4%	
75–84	300, 19.1%	126, 25.2%	
≥85	47, 3.0%	43, 8.6%	
Female	822	263	0.914
(*n*, %)	52.3%	52.6%	
AD8 score (median, IQR^&^)	0, (0, 1)	3, (2, 4)	<0.001^*※*^
^*^AD8-1 (*n*, %)	21, 1.3%	137, 27.4%	<0.001
^*^AD8-2	111, 7.1%	240, 48.0%	<0.001
^*^AD8-3	87, 5.5%	254, 50.8%	<0.001
^*^AD8-4	8, 0.5%	129, 25.8%	<0.001
^*^AD8-5	25, 1.6%	180, 36.0%	<0.001
^*^AD8-6	9, 0.6%	102, 20.4%	<0.001
^*^AD8-7	17, 1.1%	192, 38.5%	<0.001
^*^AD8-8	227, 14.5%	422, 84.4%	<0.001

^#^Defined as a total AD8 score ≥2.

^*※*^Obtained by the Wilcoxon rank sum test.

^&^IQR: interquartile range.

^*^“Change” in each AD8 subitem (number and percentage in that area).

AD8-1: problems with judgment; AD8-2: reduced interest in hobbies/activities; AD8-3: repeating questions, stories, or statements; AD8-4: difficulty in learning how to use a tool, appliance, or gadget; AD8-5: forgetting the correct month or year; AD8-6: difficulty in handling complicated financial affairs; AD8-7: difficulty in remembering appointments; AD8-8: consistent problems with thinking and/or memory.

**Table 3:** AD8 subitems based on age groups among the suspected dementia^#^ participants.

Age group years	<65 (*N* = 888)	65–74 (*N* = 667)	75–84 (*N* = 426)	≥85 (*N* = 90)	*P* value
Number (*n*, %^§^)	169 18.9%	162 24.3%	126 29.6%	43 47.8%	
Sex (female, %^⊚^)	99 58.9%	83 51.2%	57 45.2%	24 55.8%	0.124
AD8 score (median, IQR^&^)	3, (2, 4)	2, (2, 4)	3, (2, 4)	4, (2, 5)	0.005^*※*^
^*^AD8-1	47, 28.0%	38, 23.6%	33, 26.2%	18, 41.9%	0.016
^*^AD8-2	81, 48.5%	75, 46.3%	56, 44.4%	28, 65.1%	0.385
^*^AD8-3	73, 43.7%	83, 51.6%	68, 54.4%	30, 69.8%	<0.013
^*^AD8-4	34, 20.4%	43, 26.5%	36, 28.6%	16, 37.2%	0.002
^*^AD8-5	50, 29.8%	57, 35.2%	56, 44.4%	17, 39.5%	0.220
^*^AD8-6	37, 22.2%	28, 17.3%	23, 18.3%	14, 32.6%	<0.001
^*^AD8-7	57, 33.9%	59, 36.7%	54, 42.9%	22, 51.2%	0.018
^*^AD8-8	142, 84.5%	141, 87.0%	108, 85.7%	30, 69.77%	<0.001

^#^Defined as a total AD8 score of ≥2.

^§^Suspected dementia participants/total participants in this age group.

^⊚^ Female suspected dementia participants/suspected dementia participants in this age group.

^&^IQR: interquartile range.

^*※*^Obtained by the Kruskal-Wallis test.

^*^“Change” in each AD8 subitem (number and percentage in that area).

AD8-1: problems with judgment; AD8-2: reduced interest in hobbies/activities; AD8-3: repeating questions, stories, or statements; AD8-4: difficulty in learning how to use a tool, appliance, or gadget; AD8-5: forgetting the correct month or year; AD8-6: difficulty in handling complicated financial affairs; AD8-7: difficulty in remembering appointments; AD8-8: consistent problems with thinking and/or memory.
